# Extended-spectrum β-lactamase and carbapenemase producing *Enterobacteriaceae* among patients suspected with surgical site infection at Hospitals in Southern Ethiopia

**DOI:** 10.3389/fmicb.2024.1417425

**Published:** 2024-11-11

**Authors:** Desta Odoko, Abera Kumalo, Getachew Alemu, Tigistu Demisse, Teshale Mulugeta, Muluneh Temesgen

**Affiliations:** ^1^Medical Laboratory Science Department, Sodo Christian General Hospital, Sodo, Ethiopia; ^2^School of Medical Laboratory Sciences, College of Health Sciences and Medicine, Wolaita Sodo University, Sodo, Ethiopia; ^3^Department of Medical Laboratory Science, Hosanna Health Science College, Hosanna, Ethiopia

**Keywords:** ESBL, carbapenemase, *Enterobacteriaceae*, SSI, southern Ethiopia

## Abstract

**Background:**

Extended-spectrum β-lactamase and carbapenemase-producing *Enterobacteriaceae* are an increasing problem for patients today. Data on clinical samples for ESBL and carbapenemase-producing *Enterobacteriaceae* for surgical site infection patients in developing countries are limited, including Ethiopia, mainly due to resource constraints. Hence, this study aimed to determine the prevalence of extended-spectrum beta-lactamase- and carbapenemase-producing *Enterobacteriaceae* among patients suspected to have surgical site infection at Hospital in Southern Ethiopia.

**Materials and methods:**

A hospital-based cross-sectional study was conducted on 422 suspected surgical site infections from June 1, 2022 to August 30, 2022 at Hospitals in Southern Ethiopia. Sociodemographic and clinical data were obtained by using a structured questionnaire. Clinical samples (pus, pus aspirates, and wound swabs) were collected aseptically and processed within 30 min by placing the swabs in sterile test tubes containing sterile normal saline (0.5 mL). Samples were cultured on blood and MacConkey agar plates. All positive cultures were characterized by colony morphology, Gram staining, and standard biochemical tests. Antimicrobial sensitivity tests were performed using Kirby Baur disk diffusion on Mueller–Hinton agar. ESBL production was confirmed using a double-disc synergy test (DDST) method. Carbapenemase production was assessed using the modified Hodge test. Logistic regression analysis was used to determine associated factors. A *P*-value < 0.05 were considered statistically significant.

**Result:**

Bacteria belonging to the order *Enterobacterales* were cultured in 23.7% out of 422 patients with suspected surgical wound infection. Of all the isolates, *Enterobacteriaceae* (69 isolates) were the most frequent, with *E. coli* (29/69) followed by *K. pneumoniae* (14/69). Of 69 *Enterobacteriaceae* isolates, 66.6 % (46/69) were positive for ESBL production, and 21.7 (15/69) were positive for carbapenemase-producing *Enterobacteriaceae*. The majority of *Enterobacteriaceae* isolates showed sensitivity to meropenem (72.1%); however revealed 63.9% and 70.5% were resistant to gentamicin and ciprofloxacin, respectively. Similarly, a higher resistance rate to cefepime (91.8%), amoxicillin-clavulanic acid (98.4%), ceftriaxone (95.1%), and ceftazidime (91.8%). MDR rate of *Enterobacteriaceae* isolates was 25/61 (41%) among patients suspected for surgical site infection. The Multivariable analysis revealed that length of hospital stay in hospital [AOR = 3.81 (95% CI 2.08–6.95)] remained statistically significant factor associated with surgical site infection due to ESBL producing *Enterobacteriaceae*.

**Conclusion:**

Study results showed the severity of ESBL-producing *Enterobacteriaceae* is critical and CPE is alarming. Meropenem is the most effective antibiotic against the ESBL-producing *Enterobacteriaceae*. MDR rate of *Enterobacteriaceae* isolates was 61 (61%) among patients suspected for surgical site infection. Therefore, antibiotic selection should be based on the results of the culture and sensitivity tests.

## Introduction

A surgical site infection (SSI) is an infection that develops in the part of the body where the surgery is performed (Jolivet et al., 2018). Surgical site infections are among the most frequent hospital-acquired infections among surgical patients, increasing morbidity, mortality, and expense. Infections from the surgical site may be superficial infections confined to the skin. More serious infections that arise from the surgical site can affect tissues beneath the skin, organs, or implanted materials (CDC et al., 2022).

Members of the *Enterobacteriaceae* family of gram-negative bacteria produce a class of enzymes known as Extended-spectrum β-lactamases, they are complex, varied and are located on plasmids that facilitated their dissemination (Jolivet et al., 2018; CDC et al., 2022). Conversely, all *Enterobacteriaceae* that are resistant to carbapenem antibiotics (apart from those with intermediate resistance) are referred to as carbapenemase-producing *Enterobacteriaceae* (CPE). These bacteria can acquire resistance genes to tetracycline, aminoglycosides, trimethoprim, sulfonamides, and chloramphenicol through plasmids and transposons, which are essential for the development of multidrug resistance (MDR) in these bacteria (Tilahun, 2022; Ghafourian et al., 2014; Cantón et al., 2008).

According to the Health Care-Associated Infection (HAI) prevalence survey conducted by the Centers for Disease Control (CDC), there were an estimated 110,800 surgical site infections (SSIs) in 2015 that were linked to inpatient surgeries. In 2020, all National Healthcare Safety Network (NHSN) categories for surgical procedures reported a 5% decrease in the surgical site infection standardized infection ratio (SIR) compared to the year before, according to the results of the 2020 HAI data presented in the National Healthcare Safety Networks progress report (Batchelor et al., 2008).

While there have been advancements in infection prevention and control practices, such as better ventilation of surgical sites, surgical techniques, barriers, and the availability of antimicrobials or antibiotics used for prophylaxis, surgical site infections (SSIs) still account for a major cause of prolonged hospital stays, morbidity, and mortality; a study report indicates that 20% of all HAIs are caused by SSI and are associated with a 2- to 11-fold increased risk of mortality, with 75% of SSI-associated deaths being directly related to SSI (Van Duin and Doi, 2017).

On the other hand, *Enterobacteriaceae* that produce ESBL have been recognized as major bacteria that led to multidrug resistant can causing serious community-acquired and hospital-acquired infections worldwide (Ban et al., 2017). Advanced drug resistance studies recently have publicized that the emergence of ESBL-producing *Enterobacteriaceae* has increased over time. As β-lactamase-producing *Enterobacteriaceae* are not always detected in routine antimicrobial susceptibility testing, they typically demonstrate multidrug resistance. Members of the *Enterobacteriaceae* family, such as *Escherichia coli, Klebsiella pneumoniae* and other lactose non-fermenting gram-negative rods, such as *Pseudomonas* species and *Acinetobacter* spp. are important pathogens that can cause surgical site infections (Van Duin, 2017).

Global studies indicate that between 2009 and 2010, *K. pneumoniae* and *E. coli* that produce ESBL in Europe was 38.9 and 17.6%, respectively (Moges et al., 2019). In North America, the similar frequency has been estimated at 8.8 and 8.5%, respectively (Sangare et al., 2015), whereas, the prevalence in China ranged from 61 to 67% respectively. The prevalence of ESBLs in Africa, varies widely: Sudan, 30.2% (Hoban et al., 2012), Tanzania 77.1% (Saravanan et al., 2018), Algeria 47.6% (Nelson et al., 2014), Nigeria 10.3–27.5% (Nedjai et al., 2013), and from South Africa 8.8–13.1% (Hello et al., 2010), and 37.4% of isolates were ESBL positive in various samples collected from hospitals and communities in Kenya (Storberg, 2014).

Several studies in Ethiopia, have documented the range of ESBL patterns of bacteria causing site infection, there are substantial changes in the resistance profiles and etiology of bacterial pathogens across geographical areas and time (Kumalo et al., 2023). A recent report from Addis Ababa, Ethiopia: (84.1%; Beyene et al., 2019), Gondar, northwestern Ethiopia: (83.9%; Dessie et al., 2016), and Jimma southwest Ethiopia: (87.3%; Mama et al., 2014) confirmed that there is a high prevalence of ESBL producing *Enterobacteriaceae*.

However, the extensive use of antibiotics, together with the length of time over which the drugs are available in the market, has led to major problems with the emergence of resistant organisms (Mohammed et al., 2017; Temesgen et al., 2023). The issue of ESBL in developing countries has been made severe by the overuse of antibiotics, overdosing, self-medication, prescribing medications without conducting the proper susceptibility testing, and extended hospital stays. Yet, research on ESBL and CPE for SSI is lacking in developing nations like Ethiopia, and no study has previously examined the prevalence of ESBL and CPE with SSI in the southern Ethiopian region of Wolaita. Therefore, this study was aimed to determine the prevalence of extended-spectrum beta-lactamase- and Carbapenemase-producing *Enterobacteriaceae* among patients suspected to have surgical site infection at Hospital in Southern Ethiopia.

## Methods and materials

### Study design, period, and area

A hospital-based cross-sectional study was conducted from June 1, 2022 to August 30, 2022. The study was conducted at Wolaita Sodo Comprehensive Specialized Hospital (WSUCSH) and Sodo Christian General Hospital (SCGH) of the Sodo Town, Southern Ethiopia. Sodo Town is found in Wolaita zone and it is the regional cities of the Southern Ethiopia Region. The town is 329 km from Addis Ababa, the capital city of Ethiopia, and 154 km from Hawassa which is the former capital city of the region South Nation Nationalities and People Region. The WSCSH is the one of the largest hospitals in the area, which was established in 1928. It grew from District Hospital to Zonal Hospital in Wolaita Zone, town of Sodo, formerly named Wolaita Sodo Zone Hospital, until June 30, 2004, and then incorporated Wolaita Sodo University, currently serving more than 5 million people per year around the area, and also it is taught under graduating and post-graduating students. Also Sodo Christian General Hospital was established in 1997 as a primary hospital and it serves more than 4.2 million people per year including the surrounding zones and regions. Generally both hospitals have its own main mission, including quality service and providing teaching, preventive, curative care and other services in in different outpatient and inpatient departments.

### Study population

Patients who were clinically suspected of having surgical site infection.

### Sample size determination and sampling technique

#### Sample size determination

The sample size was calculated by using single population proportion formula so, considering 95% confidence interval (CI), 5% margin of error, and 50% proportion, and observed for evidence of infection on a daily basis. The clinical criteria for surgical site infection development (deep incisional surgical site infection, organ/space any surgical site infection, and superficial incisional surgical site infection) from any surgical site infection categorization system from the CDC guidelines were used (Khan and Khalil, 2011).


$$\begin{array}{l} \text{n}=\frac{\text{Z}{\text{α}}^{2}\;*\text{P}\left(1-\text{P}\right)}{{\text{d}}^{2}} \end{array}$$

By Using a 95% confidence level, the Z value was 1.96, 5% margin of error (d).


$$\begin{array}{l} \frac{{\left(1.96\right)}^{2}\;\left(0.5\;\left[1-0.5\right]\right)}{{\left(0.05\right)}^{2}}=384 \end{array}$$

Therefore, by considering a 10% contingency the final sample size was: *n* = 422.

#### Sampling technique

The study participants were enrolled using a systematic sampling technique until a sample size of 422 was achieved. The first participant was selected using the lottery method and the formula K = N/n. Thus, the Kth value for the total sample size of 422 was 1,398/422= 3. The final 422 study participants were allocated proportionally to the size of each health facility based on patient flow, as shown below. The patient flow rates for both hospitals during the previous year, 2021, were presented below.

- ***n***_WSCSH_ **=** number of sample required from Wolaita Sodo Comprehensive Specialized Hospital.
- ***N***_WSCSH_ **=** is number of SSI Patient who came to WSUCH from June 1, 2021 to August 30, 2021 GC were 728.

○ ***n***_SCGH_ **=** number of sample required from Sodo Christian General hospital.
○ ***N***_SCGH_ **=** is number of SSI Patient who came to Sodo Christian General hospital from June1, 2021 to August 30, 2021GC were 670.
▪ ***n*** **=** is the total sample size of the study was 422.

***N***_Total_ **=** the sum of SSI patients who came for service from both hospitals from June 1, 2021, to August 30, 2021, which was 1,398.

Therefore,

- ***n***_WSCSH_ (from Wolaita Sodo Comprehensive Specialized Hospital) = **(n/N**_Total_**)** ^*^ **N**_WSCSH._

○ ***n***_WSCSH_ = (422/1,398)^*^728 = **220**, sample size were allocated to WSUCH.

Whereas

- ***n***_SCGH_ (from Sodo Christian General Hospital) = **(n/N**_Total_**)** * **N**_SCGH._

○ ***n***_SCGH_= (422/1,398) × 670 = **202** was allocated to SCGH.

### Methods and tools of data collection

The participants provided with written consent, and their socio demographic and clinical data were collected using a pre-tested structured questionnaire. To ensure consistency, the questionnaire was first created in English, translated into Amharic, and then back into English. The patients' socio demographic information (age, sex, marital status, educational attainment, and employment status) and clinical information (such as antibiotic use, history of chronic illnesses, and duration of hospital stay, steroid treatment, and surgical history) were gathered via a questionnaire. Additionally, wound swabs and aspirates were obtained for laboratory analysis. Nurses and laboratory technologists from WSCSH and SCGH trained the data collectors. An extensive review of the literature was carried out (Beyene et al., 2019; Dessie et al., 2016; Mama et al., 2014).

#### Specimen collection procedure and transportation

Swabs and aspirates were collected from study participants suspected of having SSI by nurses (for swabs) and clinicians (for aspirates if suspected to be related to SSI). Sterile cotton swabs were rotated on a 1 cm^2^ area of clean granulation tissue for a period of 5 s using gentle pressure to release tissue exudate (Miller et al., 2018; National and Pillars, 2010). The fluid aspirates were collected using a 5 ml syringe. The collected specimens were transported to the Sodo Christian General Hospital Microbiology Laboratory for processing in sterile test tubes within 30 min. During the delay, Ameis transport medium was used. For carbapenemase-producing organisms, the stored organisms were transported to the Ethiopia Public Health Institute using a triple packaging system for confirmation after preserving the organisms for long-term storage (Farzana et al., 2013).

#### Identification of bacterial isolates

**Figure 1:**
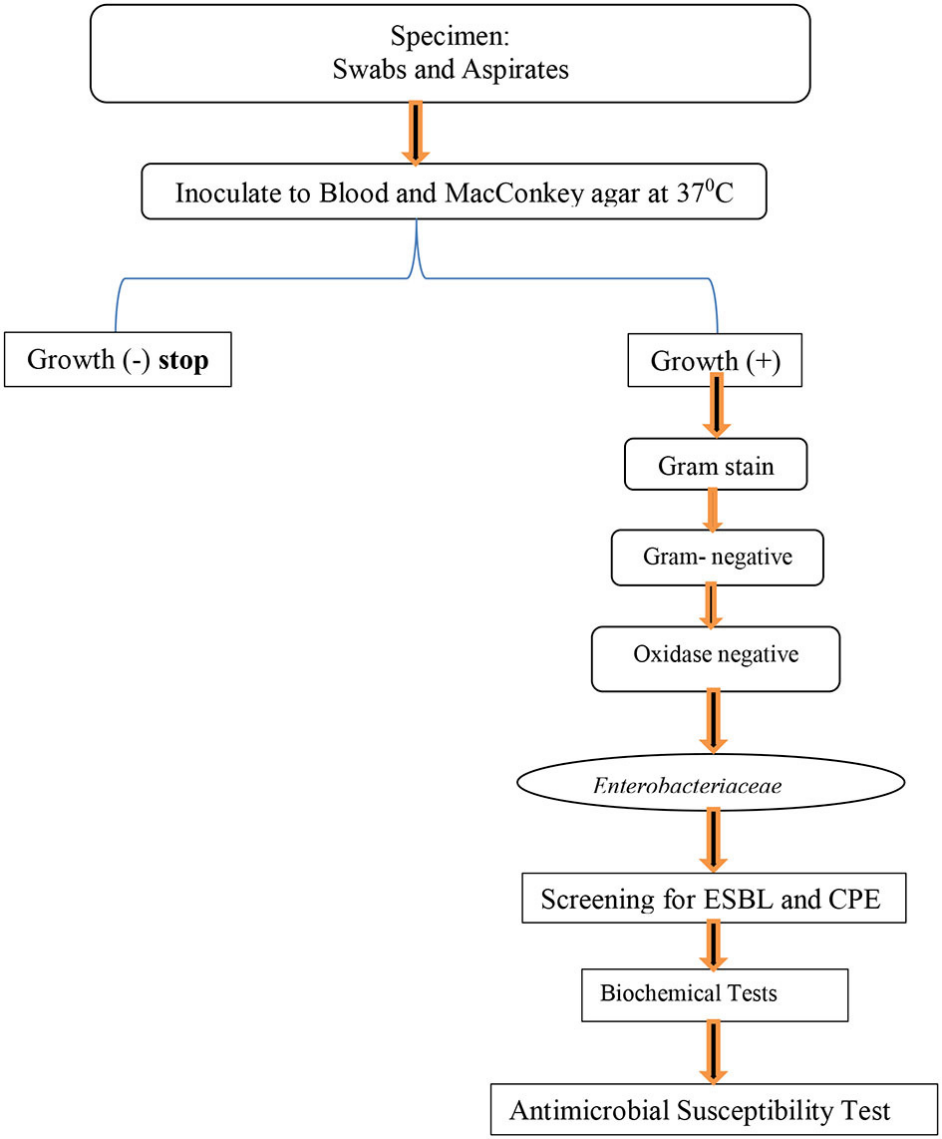
Flow chart for identification, AST, ESBL detection, and carbapenemase formation test for isolated bacterial at Hospitals in Southern Ethiopia, from Jun 1, 2022 to August 30, 2022.

All collected specimens were inoculated onto primary isolation culture media like blood agar (Oxoid Ltd, UK) and MacConkey agar (Oxoid Ltd, UK) found in Figure 1. Both blood agar (Oxoid Ltd, UK) and MacConkey agar (Oxoid Ltd, UK) were incubated at 37°C for 24–48 h. Positive cultures were inspected for growth characteristics and Gram staining was performed. Biochemical tests, such as the oxidase test, lysine and ornithine decarboxylase test, hydrogen sulfide, indole production, Vogues Proskauer (VP), urease, phenylalanine deaminase, citrate utilization, sugar fermentation from glucose and gas production, adonitol, lactose, arabinose, sorbitol, and dulcitol fermentation, were performed on colonies from primary cultures for the final identification of the isolates.

### Antimicrobial susceptibility of the isolated bacteria

Antimicrobial susceptibility testing was performed on Muller-Hinton agar (Oxoid Ltd, UK) using Modified Kirby-Bauer disc diffusion. Pure bacterial colonies were resuspended in 3–4 ml of saline solution until reaching a turbidity equivalent to 0.5 McFarland. This bacterial suspension was inoculated onto Mueller-Hinton agar plates using cotton swabs (Weinstein, 2021). The following antibacterial agents were selected based on local prescription habits and CLSI recommendations. The standard antibiotic disks (Thermo Fisher Scientific, USA) and their concentrations were as follows: Gentamicin GEN 10 μg, Chloramphenicol CHO 30 μg, Ciprofloxacin CIP 5 μg, Amoxicillin-clavulanic acid AUG (25+30) μg), Meropenem MER 10 μg, Cefepime CEF 30 μg, Ceftazidime CAZ 30 μg, ceftriaxone CRO 30 μg, and Cefixime CFM 5 μg. The diameter of the inhibition zone around the disc was measured with a ruler in millimeters and interpreted as sensitive, intermediate and resistant according to Clinical Laboratory Standards Institute (Weinstein, 2021; Carvalhaes et al., 2009).

### Detection of ESBL-producing *Enterobacteriaceae*

#### Double-disc synergy test method

Disks containing ceftazidime were applied next to those containing clavulanic acid and amoxicillin. Positive results were obtained when the inhibition zones around any of the cephalosporin disks was enlarged in the direction of the disc containing the clavulanic acid. The distance between the disks is critical and a 20 mm center-to-center has been found to be optimal for cephalosporin 30 μg disks (Weinstein, 2021).

### Methods for confirmation of carbapenemase-producing *Enterobacteriaceae*

#### Modified Hodge test

The Modified Hodge Test (MHT) is a simple phenotypic test and has been suggested as a method used for screening carbapenemases in bacteria. It is based on the inactivation of carbapenem by carbapenemase-producing strains, that is, test isolates that enable a carbapenem-susceptible indicator strain *E. coli* ATCC 25922 to extend growth toward a carbapenem-containing disc along with the streak of the inoculum of the test strain. A positive test result indicated a clover leaf-like indentation (Cury et al., 2012; Date, 2018; Pierce et al., 2017; Reumert et al., 2003; Giske and Cantón, 2013).

*Enterobacteriaceae* suspected to be producers of carbapenemase were confirmed 10 μg meropenem disc (MRP) and by evaluating the synergistic effects when combined with the following inhibitors: phenylboronic acid, DPA/EDTA and Cloxacilline (Giske et al., 2011; Tsakris et al., 2010). The inhibition zone around the meropenem disc combined with the inhibitor was compared with the zone around the disc (Alebel et al., 2021).

### Laboratory quality assurance

The sample quality was examined and handled in accordance with each hospital's quality control protocols. Using sterile cotton, skilled staff nurses aseptically removed pus swabs representative of the infected tissue from the affected area. The pus swabs were then cleaned with an antiseptic solution. As soon as possible, the gathered specimens were moved to the Sodo Christian General Hospital Microbiology Laboratory, and the specimens that were delayed were put in the Amies transport medium. Standard operating procedures were followed when performing laboratory analyses (SOPs). In order to confirm the sterility of the culture media, 5% of the prepared media were incubated overnight before being inoculated with the standard reference strains of *E. coli* (ATCC 700682) used to check of ESBLs (ATCC 25922), used as negative control of ESBLs, *K. pneumoniae* (ATCC 13883 and *Klebsiella quasipneumoniae* formerly known as *K. pneumoniae (*ATCC 700603) were used for the positive and negative control, respectively.

### Data processing, analysis, and interpretation

Data were entered, checked, cleaned, coded for completeness in Epi 4.6.0.2, and imported into the Statistical Package for Social Sciences (SPSS) software version 25 for analysis. Descriptive statistics were computed, summarized, and presented using graphs and tables. Binary logistic regression was used to examine the relationship between dependent and independent variables. Statistical significance was set at *P* < 0.05.

## Results

### The study participants' socio-demographic characteristics

With a 100% response rate, 422 patients across all age groups who had a suspicion of SSI were included in this study found in Table 1. Two-hundred and thirty-five (55.7%) of the study groups' members were female. With an age range of 5–96 years, the participants with the highest number, 224 (53.1%), were between the ages of 31 and 44. The majority of participants, or 303 (71.8%), were from urban areas; 123 (52.1%) were students, 69 (16.4%) were government workers, 31 (98.3%) were merchants, 84 (19.9%) were college graduates or above, and 277 (67.1%) were married.

**Table 1:** Socio-demographic characteristics of patients with suspicion of SSI at Hospitals in Southern Ethiopia, from Jun 1, 2022 to August 30, 2022 (*n* = 422).

**Variables**		**Frequency**	**Percent (%)**
Sex	Male	187	44.3
	Female	235	55.7
Age group	< 18	7	1.7
	18–30	55	13.0
	31–44	224	53.1
	45–59	96	22.7
	60–75	29	6.9
	>75	11	2.6
Residence	Rural	119	28.2
	Urban	303	71.8
Marital status	Single	133	31.5
	Married	289	68.5
Educational status	Illiterate	59	14.0
	Read and write	82	19.4
	Primary education	66	15.6
	High school	131	31.1
	Diploma and above	84	19.9
Occupational	Farmer	39	9.2
	Governmental employee	69	16.4
	Student	123	52.1
	Daily labor	56	13.2
	House wife	104	91.0
	Merchant	31	98.3

### Clinical and medical status of the study participants

A total of 422 participants were admitted to the study. Of these, 221 (52.4%) were from the orthopedics ward, 112 (26.5%) from the surgery ward, and 89 (21.1%) from the Obstetrics and Gynecology Unit. A significant percentage of the participants (44.3%) had clean wounds. The largest number of days spent in the hospital was 8–14 days (34.8%), and there were 27 (6.4%) diabetics overall (Table 2).

**Table 2:** Clinical and medical status among patients with suspicion of SSI at Hospitals in Southern Ethiopia, from Jun 1, 2022 to August 30, 2022 (*n* = 422).

**Variables**		**Frequency**	**Percent (%)**
Surgical site wards	Orthopedic	221	52.4%
	Surgical	112	26.5%
	Gynecology and obstetrics	89	21.1%
Wound types	Non-contaminated	187	44.3%
	Clean-contaminated	123	29.1%
	Contaminated	54	12.8%
	Infected	58	13.7%
Chronic liver disease	Yes	2	0.5%
	No	420	99.5%
Heart failure	Yes	5	1.2%
	No	417	98.8%
Diabetes	Yes	27	6.4%
	No	395	93.6%
HIV/AIDS	Yes	11	2.6%
	No	411	97.4%
Immunosuppressive (long term steroid)	Yes	28	6.6%
	No	394	93.4%
Travel history to abroad	Yes	14	3.3%
	No	408	96.7%
Length of medical stay in hospital	1–7 days	137	32.5
	8–14 days	147	34.8
	>15 days	138	32.7

### Distribution of *Enterobacterales* isolates among patients suspected for surgical site infection

Four hundred and twenty-two wound swabs in total were used to cultivate *Enterobacterales* (family *Enterobacteriaceae* and *Morganellaceae)*. One hundred out of the suspects exhibited both *Enterobacteriaceae* and *Morganellaceae* growth. Significant *Enterobacterales* infections were present in 23.7% (100/422) of patients suspected of having surgical site infections. From the total isolates, 69 (69%) strains belong to the family *Enterobacteriaceae* includes *E. coli* 29 (29%), followed by *K. pneumoniae* 14 (14%), was the most common isolate. And also, 31(31%) strains considered to be the family *Morganellaceae* (*M. morganii, Providence* spp., and *P. mirabilis*) were isolated (Table 3).

**Table 3:** Identified *Enterobacterales* isolates among patients suspected for surgical site infection at Hospitals in Southern Ethiopia, from Jun 1, 2022 to August 30, 2022 (*n* = 100).

**Bacterial isolates**	**Percent (%)**
*E. coli*	29
*K. pneumonia*	14
*K. oxytoca*	8
*E. clocae*	6
*C. freundii*	4
*M. morganii*	10
*P. mirabilis*	10
Providencia spp.	11
*E. aerogenes*	8
Total	100

### Antimicrobial susceptibility pattern of extended-spectrum β-lactamase and carbapenemase producing *Enterobacterales*

In present study (Table 4), the higher resistance rate to cefepime reported by the majority of Enterobacteriaceae isolates (91.8%), ceftriaxone (95.1%), ceftazidime (91.8%), and amoxicillin-clavulanic acid (98.4%). However, Enterobacteriaceae isolates showed relatively high sensitivity to Meropenem 44 (72.1%). On the other hand, high percentages of resistance were obtained, gentamicin: 39 (63.9%) and ciprofloxacin: 43 (70.5%), respectively. The isolates of K. pneumoniae is resistant to cefepime, Ceftriaxone, Ceftazidime, Amoxicillin-clavulanic acid, Gentamicin, ciprofloxacin and meropenem; 14 (100%), 14 (100%), 14 (100%), 14 (100%), 12 (85.7%), 11 (78.6%), and 9 (64.3%), respectively. And also, isolates of C. freundii resistant to cefepime, ceftriaxone, ceftazidime, amoxicillin-clavulanic acid was 4 (100%) by each, and gentamicin and chloramphenicol was 3 (75%) by each, similarly, the resistance of E.coli species to cefepime, Ceftriaxone, Ceftazidime, Amoxicillin-clavulanic acid and ciprofloxacin, 26 (90%), 27 (93.2%), 26 (90%), 28 (96.6%), and 22 (75.9%), respectively. On the other hand, sensitivity rates for chloramphenicol 7(87.5%) and meropenem 6(75%) were highest for K.oxytoca. The susceptibility patterns of K. pneumoniae to gentamicin was 2(14.4%), ciprofloxacin 3 (21.4%), chloramphenicol 6 (42.8%), and meropenem 5 (35.7%) that was < 50%.

**Table 4:** Antimicrobial susceptibility pattern of extended-spectrum β-lactamase and carbapenemase producing *Enterobacterales* isolates (*n* = 100) among surgical site infection at Hospitals in Southern Ethiopia, from Jun 1, 2022 to August 30, 2022 (*n* = 100).

***Enterobacterales* isolated**		**Antimicrobials tested for** ***Enterobacteriaceae*** **isolates**							
		**CEP**	**CRO**	**CAZ**	**AMC**	**CIP**	**GEN**	**CHL**	**MEM**
*K. pneumoniae* (*n* = 14)	S	0 (0%)	0 (0%)	0 (0%)	0 (0%)	3 (21.4%)	2 (14.3%)	6 (42.8%)	5 (35.7%)
	R	14 (100%)	14 (100%)	14 (100%)	14 (100%)	11 (78.6%)	12 (85.7%)	8 (57.2%)	9 (64.3%)
*E. coli* (*n* = 29)	S	3 (10%)	2 (6.8%)	3 (10.3%)	1 (3.4%)	7 (24.1%)	16 (55.2%)	21 (72.4%)	25 (86.2%)
	R	26 (90%)	27 (93.2%)	26 (89.7%)	28 (96.6%)	22 (75.9%)	13 (44.8%)	8 (27.6%)	4 (13.8%)
*K. oxytoca* (*n* = 8)	S	1 (12.5%)	1 (12.5%)	1 (12.5%)	0 (0%)	3 (37.5%)	1 (12.5%)	7 (87.5%)	6 (75%)
	R	7 (87.5%)	7 (87.5%)	7 (87.5%)	8 (100%)	5 (62.5%)	7 (87.5%)	1 (12.5%)	2 (25%)
*E. clocae* (*n* = 6)	S	1 (16.7%)	0 (0%)	1 (16.7%)	0 (0%)	3 (50%)	2 (33.3%)	3 (50%)	6 (100%)
	R	5 (83.3%)	6 (100%)	5 (83.3%)	6 (100%)	3 (50%)	4 (66.7%)	3 (50%)	0 (0%)
*C. freundii* (*n* = 4)	S	0 (0%)	0 (0%)	0 (0%)	0 (0%)	2 (50%)	1 (25%)	1 (25%)	2 (50%)
	R	4 (100%)	4 (100%)	4 (100%)	4 (100%)	2 (50%)	3 (75%)	3 (75%)	2 (50%)
*M. morganii (n=10)*	S	_	_	_	_	_	_	_	_
	R	_	_	_	_	_	_	_	_
*P. mirabilis* (*n* = 10)	S	_	_	_	_	_	_	_	_
	R	_	_	_	_	_	_	_	_
*Providencia* spp. (*n* = 11)	S	_	_	_	_	_	_	_	_
	R	_	_	_	_	_	_	_	_
*E. aerogenes* (*n* = 8)	S	_	_	_	_	_	_	_	_
	R	_	_	_	_	_	_	_	_
Total (100)	S	5 (8.2%)	3 (4.9%)	5 (8.2%)	1 (1.6%)	18 (29.5%)	22 (36.1%)	38 (62.3%)	44 (72.1%)
	R	56 (91.8%)	58 (95.1%)	56 (91.8%)	60 (98.4%)	43 (70.5%)	39 (63.9%)	23 (37.7%)	17 (27.9%)

R, Resistant; S, Sensitive, CEP, Cefepime, CRO, Ceftriaxone, CAZ, Ceftazidime, AMC, Amoxicillin-clavulanic acid; CIP, Ciprofloxacin; GEN, Gentamicin; MEM, Meropenem; CHL, Chloramphenicol; (_), not tested.

### Prevalence of extended-spectrum β-lactamase and carbapenemase producing *Enterobacterales*

**Figure 2:**
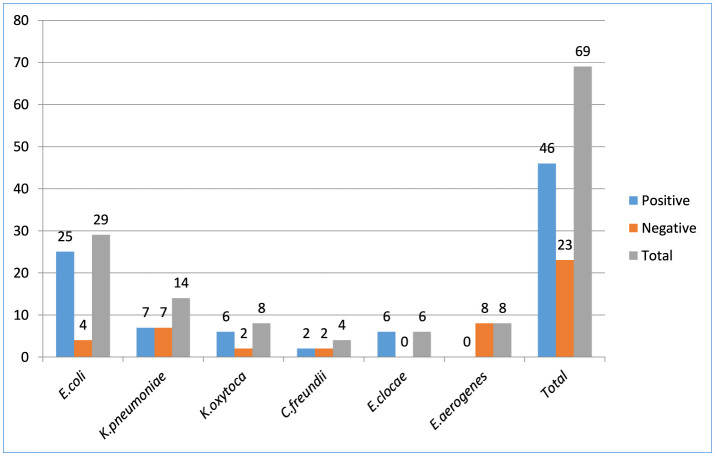
*Enterobacteriaceae* bacterial isolates producing extended-spectrum β-lactamase among patients suspected for surgical site infection at Hospitals in Southern Ethiopia, from Jun 1, 2022 to August 30, 2022 (*N* = 422).

From wound swabs, 100 and 61 different *Enterobacterales* and *Enterobacteriaceae* were identified, respectively. Forty-six (66.6%), out of the 69 *Enterobacteriaceae* isolates had their ESBL production confirmed phenotypically. From all isolates of *Enterobacteriaceae* that were suspected producing ESBLs, three *Enterobacteriaceae* isolates were ESBLs tested positive: *E. coli* (54.35%, *n* = 25/46), *K. pneumoniae* (15.2%, *n* = 7/46), *K. oxytoca* (13.04%, *n* = 6/46), *C. freundii* (4.35%, *n* = 2/46), *E.clocae* (13.04%, *n* = 6/46), and *E. aerogenes* (0%, *n* = 0/46). The proportion of ESBL *Enterobacterales* isolates overall was 46% (46/100; Figure 2).

**Figure 3:**
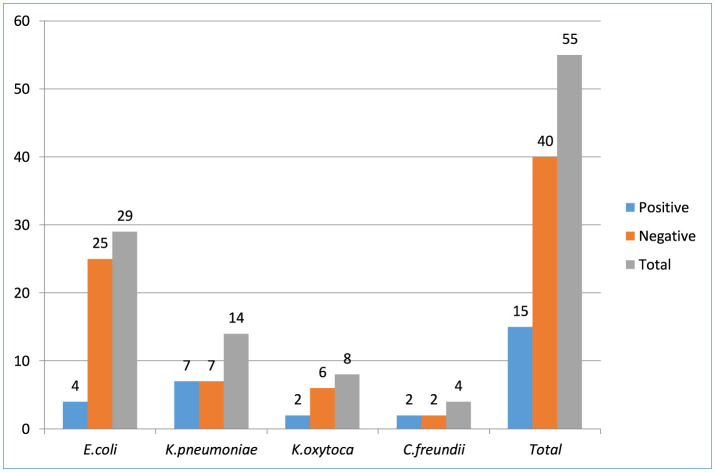
*Enterobacteriaceae* bacterial isolates producing carbapenemase among patients suspected for surgical site infection at Hospitals in Southern Ethiopia, from Jun 1, 2022 to August 30, 2022 (*N* = 422).

Carbapenemase production was tested against all *Enterobacterales*, regardless of the ESBL results, and 21.7 % (15/69) isolates had carbapenemase production confirmed. Among tested isolates resistant to carbapenem, *K. pneumoniae;* 7/15 (46.7%), *E. coli;* 4/15 (26.7%), *K. oxytoca;* 2/15 (13.3%), and *C. freundii;* 2/15 (13.3%) were the most common *Enterobacteriaceae*. The overall proportion of *Enterobacterales* isolates that producing carbapenemase were 15% (15/100) (Figure 3).

#### Class carbapenemase producing Enterobacterales

Out of fifteen distinct *Enterobacterales* isolates that were thoroughly examined and were thought to be non-repeats, seven (*K. pneumoniae*), four (*E. coli*), two (*C. freundii*), and two (*K. oxytoca*) tested positive for carbapenemase production. Specifically, carbapenemases were not detected in 80% of the carbapenem-resistant strains. In this group, AmpC plus porin loss was detected. Only in 3 strains were these enzymes detected (2 NDM and 1 KPC). AmpC plus porin loss 12 (80%), KPC plus MBL 1 (6.7%), and MBL 2 (13.3%) were the results of the isolates.

### Multidrug-resistant pattern of extended-spectrum β-lactamase and carbapenemase producing *Enterobacterales*

The total *Enterobacterales* isolated from patients suspected for surgical site infections, MDR rate were 25/61 (41%) found in Table 5. Approximately 41.0% of *Enterobacterales* isolates were MDR, 47.5% XDR, and 4.9% PDR isolates. Among the *Enterobacterales* isolates, *E. coli* (48.3% MDR, 37.9% XDR, and 3.4% PDR), *K. pneumoniae* (21.4% MDR, 71.4% XDR, and 7.1% of PDR), *K.oxytoca* (37.5% MDR, 50% XDR, and 12.5% PDR), *E. clocae* (66.7% MDR, 16.7% XDR), and *C. freundii* (25% MDR, 75% XDR) were developed multidrug resistant.

**Table 5:** Multidrug-resistant pattern of extended-spectrum β-lactamase and carbapenemase producing *Enterobacteriaceae* isolates among surgical site infection at Hospitals in Southern Ethiopia, from Jun 1, 2022 to August 30, 2022 (*n* = 61).

***Enterobacterales* isolates**	**MultiS**	**MoDR**	**MDR**	**XDR**	**PDR**
*K. pneumoniae* (*n* = 14)	0 (0.0%)	0 (0.0%)	3 (21.4%)	10 (71.4%)	1 (7.1%)
*E. coli* (*n* = 29)	0 (0.0%)	0 (0.0%)	14 (48.3%)	11 (37.9%)	1 (3.4%)
*K. oxytoca* (*n* = 6)	0 (0.0%)	0 (0.0%)	3 (37.5%)	4 (50.0%)	1 (12.5%)
*E. clocae* (*n* = 6)	0 (0.0%)	0 (0.0%)	4 (66.7%)	1 (16.7%)	0 (0.0%)
*C. freundii* (*n* = 4)	0 (0.0%)	0 (0.0%)	1 (25.0%)	3 (75.0%)	0 (0.0%)
Total (61)	0 (0.0%)	0 (0.0%)	25 (41.0%)	29 (47.5%)	3 (4.9%)

MultiS, susceptible to all antibiotic classes; MoDR, resistant to a single antibiotic class; MDR, resistant to at least one agent in three or more antimicrobial categories; XDR, resistant to at least one agent in all but two or fewer antimicrobial categories (i.e., bacterial isolates remain susceptible to only one or two categories); PDR, resistant to all antibiotic classes.

#### Factors association with extended-spectrum β-lactamase and carbapenemase producing Enterobacteriaceae among patients suspected for surgical site infection

Four variables with *p*-values < 0.25 in univariate analysis suggested using the multivariate model. Age ranges of 31–44 and 45–96 years, respectively, had AORs of 1.51 and 2.88 and 0.92 and 0.45–1.85, 95% confidence intervals. Individuals admitted to the orthopedic and surgical wards had AORs of 1.15 and 0.64–2.05 and 1.94 and 95% CI: 1.04–3.65, respectively, and their length of hospital stay were 8.14 days for the former and >15 days for the latter (AOR = 3.81 and 95% CI: 2.08–6.95). Candidates for multivariate logistic regression analysis were patients with diabetes and those without (AOR = 1.91 and 95% CI: 0.77–4.76), respectively. A similar independent association between the candidate variables and surgical site infection caused by *Enterobacteriaceae* that produce ESBLs and CPEs was further evaluated at a cutoff point of *P*-value < 0.05 in multivariable logistic regression analysis. Out of all of them, the multivariate logistic regression analysis revealed a significant association with only the length of hospital stay being independently associated. Length of medical stay in hospital AOR = 3.81 (95% CI 2.08–6.95) was found to be a statistically significant factor in surgical site infection caused by *Enterobacteriaceae* that produce CPE and ESBL in the multivariable analysis. Due to *Enterobacteriaceae* that produce CPE and ESBL, patients hospitalized for 8–14 days had a 6-fold higher risk of surgical site infection than patients who stayed for >15 days; the other variables were not significantly associated with this risk.

## Discussion

The main aim of this study was to address three key objectives. All these objectives determined the prevalence, antibiotic susceptibility patterns, and factors associated with Extended-spectrum β-lactamase and carbapenemase-producing *Enterobacteriaceae* among patients suspected with surgical site infection.

In the present study, the total prevalence of *Enterobacteriaceae* growth among surgical site infections was 23.7%. This finding is consistent with the previous reports in Addis Ababa, Ethiopia (25.13%) (Beyene et al., 2019), Bahir Dar, Ethiopia (24.8%) (Alebel et al., 2021), France (25%) (Baron et al., 2013), Nepal (28.2%) (Kayastha et al., 2020), Qatar (26%) (Sid Ahmed et al., 2016), and India (21.4%) (Gupta et al., 2016). However, this was lower than the research conducted in Debra Markos, Ethiopia (72.6%) (Shimekaw et al., 2022), Gondar (83.9%) (Mohammed et al., 2017), Jimma (87.3%) (Mama et al., 2014), Addis Ababa (84.1%) (Beyene et al., 2019), Nigeria (64.8%) (Egbe et al., 2011), India (68 %) (Vasundhara et al., 2017), and Nepal (65%) (Rijal et al., 2017) and higher than the study done in Mexico (19%) (Uc-Cachón et al., 2019), India (19.5%) (Singh et al., 2016), and Nepal (12.6%) (Kayastha et al., 2020). These variations among different studies could be due to variations in the study population, bad or good antibiotic stewardship policies, level of wound care, and specimen type.

In the present study, the prevalence of ESBL-producing *Enterobacterales* was 46%, which is in line with the institution-based based cross-sectional study conducted in Algeria (47.6%) (Nedjai et al., 2013), and Philippines (43%) (Bell et al., 2003). However, it was higher than that reported in Bahir Dar Ethiopia 24.8% (Alebel et al., 2021), India 35.2%(Oberoi et al., 2013), France (25%) (Baron et al., 2013), Nepal (28.2%) (Kayastha et al., 2020), Qatar (26%) (Sid Ahmed et al., 2016), and India (21.4%) (Gupta et al., 2016). In some of this study since compared to the aforementioned studies or for ESBL-producing *Enterobacteriaceae*, colistin was an alternative treatment only in the study area, however carbapenemase are not used as treatment (Katip et al., 2021).

In this study among *Enterobacteriaceae, E. coli* 29/100 (29.0%) was the predominant isolate associated with surgical site infection, followed by *K. pneumoniae* 14/100 (14%), *K. oxytoca* 8/100(8%), *E. clocae* 6/100 (6%) and *C. freundii* 4/100 (4%). This is supported by various findings in Africa, Uganda, Algeria, Brazil (Iabadene et al., 2008; Andrew et al., 2017; Ibrahim et al., 2013; Pollack and Srinivasan, 2014) and Mexico (Uc-Cachón et al., 2019). These findings revealed that *Enterobacteriaceae* isolates were the most common hospital-acquired infections.

In our study, the prevalence of carbapenemase producing Enterobacteriaceae isolates was 15%, which agreed with the research conducted in Taiwan, (15.4%) (Chang et al., 2015) Indonesia (13.7%) (Saharman and Lestari, 2013). This finding is higher than that study conducted in Bahir Dar, Ethiopia (5.2%) (Alebel et al., 2021), Addis Ababa Ethiopia (2%) (Beyene et al., 2019), and CDC reports from (2001) (1.2%) and 2011 (4.2%) (Ramana et al., 2013); however, our findings were lower than those reported in Nigeria (36%) (Pollack and Srinivasan, 2014), India (34.5%) (Oberoi et al., 2013), and 44.1% (Makharita et al., 2020). This may be attributed to the rapid expansion of CPE-producing organisms or misuse of antibiotics.

The most common carbapenemase producing Enterobacteriaceae isolates in our study were K. pneumoniae (46.7 %), followed by E. coli (26.7%) and K. oxytoca (13.3%). A similar finding in Nigeria E. coli 15.4% and K. pneumoniae 40.9% (Pollack and Srinivasan, 2014), and Tanzania E. coli (14%), followed by K. pneumoniae (10.57%) are carbapenemase-producing Enterobacteriaceae (Mushi et al., 2014). In this study, the most common carbapenemases were MBL 2 (13.3%), KPC plus MBL 1 (6.7%) and on the other side, AmpC plus porin loss 12 (80%). These findings are in line those study conducted on USA isolates produced KPC 36 (28%), MBL 31 (24.2%), KPC plus MBL 4 (3.1%), ESBL 19 (14.8), AmpC 10 (7.8%) (van Dijk et al., 2014). A similar study in India showed MBL producers were 10.98% similar to this study (Kumar et al., 2012), while AmpC production was observed in 5.4% (Oberoi et al., 2013), which is much less than our findings. This shows that in our study, plasmid and transposon-mediated gene transfer was more common in our isolates than in the Indian study. This is may be due to variation of geographical location and use of sample size amount. The coexistence of different classes of β-lactamases in a single bacterial isolate may pose diagnostic and treatment challenges, although high-level expression of AmpC β-lactamases may mask the recognition of ESBLs, which may result in more difficult antimicrobial therapy (Oberoi et al., 2013).

The current study showed that *Enterobacteriaceae* had higher antibiotic resistance rates of for cefepime (91.8%), ceftriaxone (95.1%), ceftazidime (91.8%), and amoxicillin-clavulanic acid (98.4%). However, *Enterobacteriaceae* isolates showed relatively high sensitivity to Meropenem 44 (72.1%). On the other hand, high percentages of resistance were obtained, gentamicin: 39 (63.9%) and ciprofloxacin: 43 (70.5%), respectively. This finding is consistent with that study conducted in Nepal on cefepime (86.2%), ceftriaxone (100%), and ceftazidime (100%) were resistance (Beyene et al., 2019). Similarly to studies conducted in Ethiopia, amoxicillin/clavulanic acid 75% and cefotaxime (73.7%) were found to be resistant to *Enterobacteriaceae* isolates (Dessie et al., 2016).

In the current study, MDR rate of *Enterobacteriaceae* isolates was 41% (showing resistance to at least one agent in ≥3 antimicrobial categories) among patients suspected of surgical site infection, 47.5% (showing resistance at least to one agent in all but two or fewer antimicrobial categories) XDR, and 4.9% (showing resistance to all agents in all antimicrobial categories) PDR were resistant, respectively. Among the *Enterobacteriaceae* isolates, *E. coli*, 48.3% MDR, 37.9% XDR, 3.4% PDR, *K. pneumoniae* 21.4% MDR, 71.4% XDR, 7.1% of PDR, *K. oxytoca* 37.5% MDR, 50% XDR, 12.5% PDR, *E. clocae* 66.7% MDR, 16.7% XDR, *C. freundii* 25% MDR, 75% XDR were developed resistance, while the study in Jimma, Ethiopia showed MDR 30.16%, XDR 41.27%, and PDR 19.0% were found (Mama et al., 2014; Gashaw et al., 2018), there are few previous reports from Ethiopia on XDR and PDR *Enterobacteriaceae* to compare with this finding, which has been associated with lack of AMR surveillance and stewardship programs at WSUCSH and SCGH and also in southern Ethiopia.

Factors associated with surgical site infection with ESBL and CPE-producing *Enterobacteriaceae* included indicators of contact with the health care system (length of hospital stay, immunosuppressive drug (long-term steroid; Livermore et al., 2011), patient admitted wards, educational level, and the presence of comorbidities). Four variables of associated factors were independently predictive of ESBL positivity as showed *P*-value of < 0.25 by univariate analysis: age in group 31–45 years, immunosuppressive drug use (long-term steroid), length of hospital stay, and diabetes. Among them, only the length of hospital stay was independently associated with the multivariate logistic regression analysis, which showed a significant association. This study was supported by a previous study conducted in Israel (Mendelson et al., 2005). The increased odds of our finding was that the presence of predisposing conditions such as surgical intervention was significantly associated with ESBL producers (*P* < 0.05) compared with a short length of hospital stay, which is similar to a study conducted in Debra Markos, Ethiopia (Alebel et al., 2021). In this study, the remaining variables were not significantly associated with surgical site infections caused by ESBL-producing *Enterobacteriaceae* (*P* >0.05; Table 6).

**Table 6:** Bivariate and multivariate logistic regression analysis on socio demographic and clinical condition of the patients suspected for surgical site infection at Hospitals in Southern Ethiopia, from Jun 1, 2022 to August 30, 2022 (*n* = 422).

**Variables**	**Categories**	**Confirmed surgical site infection due to ESB producing** ***Enterobacteriaceae***		**Bivariate**		**Multivariate logistic regression**	
		**Yes** ***N*** **(%)**	**No** ***N*** **(%)**	**COR (95% CI)**	***P*** **-value**	**AOR (95% CI)**	***P*** **-value**
Age in years	5–30	16 (25.8)	46 (74.2)	1.00	1.00	1.00	1.00
	31–44	72 (32.1)	152 (67.9)	1.36 (0.72,2.57)	0.120	1.51 (0.79,2.88)	0.34
	45–96	31 (22.8)	105 (77.2)	0.85 (0.42,1.70)	0.061	0.92 (0.45,1.85)	0.050
Wards	Orthopedic	56 (25.3)	165 (74.7)	1.03 (0.59,1.82)	0.048	1.15 (0.64,2.05)	0.070
	Surgical	41 (36.6)	71 (63.4)	1.76 (0.95,3.26)	0.095	1.94 (1.04,3.65)	0.105
	Obis/Gyn	22 (24.7)	67 (75.3)	1.00	1.00	1.00	1.00
Length of medical stay in hospital	1–7 days	24 (17.5)	113 (82.5)	1.00	1.00	1.00	1.00
	8–14 day	57 (38.8)	90 (61.2)	1.67 (1.01,2.75)	0.001	3.81 (2.08,6.95)^*^	**0.010**
	>15 days	38 (27.5)	100 (72.5)	1.79 (1.01,3.19)	0.045	1.83 (1.01,3.34)	**0.047**
Diabetics	Yes	13 (48.1)	14 (51.9)	2.53 (1.15,5.56)	0.020	1.91 (0.77,4.76)	0.163
	No	106 (26.8)	289 (73.2)	1.00	1.00	1.00	1.00

1.00, Reference, COR, crude odds ratio; AOR, adjusted odds ratio; CI, Confidence Interval; Oby/Gyn, Obstetrics and Gynecology.

^*^Statistical significance was set at P-value < 0.05.

### Strength and limitation of the study

This study has several advantages. It provides useful information about the prevalence and antimicrobial susceptibility trends of potentially pathogenic *Enterobacteriaceae* in hospital settings, especially in SSI cases. This study also found the presence of multidrug-resistant bacteria, including ESBLs and carbapenemase-producing strains, which are extremely dangerous in healthcare settings. Drug susceptibility testing for nine antibiotics including extended-spectrum cephalosporins; 3rd and 4th generation cephalosporins, carbapenemase plus combination disks like Meropenem 10 μg, Meropenem+Phenolboroic acid, Meropenem+Dipicolonic acid, Meropenem +Cloxacillin, and Temocillin 30 μg was tested. This study had a drawback, although it was conducted in a two hospital, which might not be an accurate representation of other hospitals in the country level. Anaerobic and slow-growing bacteria were not considered in this study. Ultimately, the results of the study were a low number of *Enterobacteriaceae* isolates, which resulted in a low number of ESBL and CPE that might have affected the findings of the risk factors. Additionally, the study was limited by the available resources and time frame for research that makes identification of ESBL- and carbapenemase-producing strains by phenotypically only even if it is recommended to perform detection by molecular biology techniques for identification of ESBL- and carbapenemase-producing strains.

## Conclusion and recommendation

*Enterobacteriaceae* were present in all SSIs at a rate of 23.7% (100/422). *E. coli* predominated, followed by *K. pneumoniae*, with a total prevalence of 46% for ESBL and 15% for CPE. It was concerning to see the high rates of MDR and XDR ESBL and carbapenem resistance. According to the study's findings, a hospital stay's duration significantly affects the risk of site infection. When making decisions about the high prevalence of MDR *Enterobacteriaceae* in relation to antibiotics like cefepime, ceftriaxone, ceftazidime, and amoxicillin-clavulanic acid, empirical treatment should be taken into account. On the other hand, thorough instruction, referral, and standard hospital procedures help avoid or manage surgical site infections. When choosing the optimal antimicrobial agents for patients with infections caused by ESBL *Enterobacteriaceae* and CRE in southern Ethiopia, physicians can make informed decisions about public health thanks to the documented data presented in this study. Therefore, in order to address this public health issue, stakeholders involved in Ethiopia's health system must work cooperatively. And also, appropriate antibiotic prescribing practices and avoidance of broad spectrum antibiotics can prevent long-term emergence of resistance. Additionally, more investigation is required to fully understand the implications of *Enterobacteriaceae* that produce CPE and ESBL and their effects on patients who may be at risk for surgical site infections in different hospital with different level.

## Data Availability

The datasets presented in this study can be found in online repositories. The names of the repository/repositories and accession number(s) can be found in the article/supplementary material.
